# Complement activating ABO anti-A IgM/IgG act synergistically to cause erythrophagocytosis: implications among minor ABO incompatible transfusions

**DOI:** 10.1186/s12967-020-02378-w

**Published:** 2020-05-28

**Authors:** Priyanka Pandey, Waseem Q. Anani, Tina Pugh, Jerome L. Gottschall, Gregory A. Denomme

**Affiliations:** 1 Diagnostic Laboratories, Versiti Blood Center of Wisconsin, 638 N 18th Street, Milwaukee, WI 53233 USA; 2 Versiti Blood Research Institute, Milwaukee, USA; 3 Department of Pathology, Medical College of Wisconsin, Wauwatosa, WI USA

**Keywords:** Anti-A, Complement C3b receptor, Fcγ receptor, Hemolysin

## Abstract

**Background:**

Typically minor ABO incompatible platelet products are transfused without any incident, yet serious hemolytic transfusion reactions occur. To mitigate these events, ABO ‘low titer’ products are used for minor ABO incompatible transfusions. We sought to understand the role of IgM/IgG and complement activation by anti-A on extravascular hemolysis.

**Methods:**

Samples evaluated included (i) Group O plasma from a blood donor whose apheresis platelet product resulted in an extravascular transfusion reaction, (ii) Group O plasma from 12 healthy donors with matching titers that activated complement (N = 6) or not (N = 6), and (iii) Group O sera from 10 patients with anti-A hemolysin activity. A flow cytometric monocyte erythrophagocytosis assay was developed using monocytes isolated by immunomagnetic CD14-positive selection from ACD whole blood of healthy donors. Monocytes were frozen at − 80 °C in 10% dimethyl sulfoxide/FBS and then thawed/reconstituted on the day of use. Monocytes were co-incubated with anti-A-sensitized fluorescently-labeled Group A1 + RBCs with and without fresh Group A serum as a source of complement C3, and erythrophagocytosis was analyzed by flow cytometry. The dependency of IgM/IgG anti-A and complement C3 activation for RBC erythrophagocytosis was studied. Anti-A IgG subclass specificities were examined for specific samples.

**Results:**

The plasma and sera had variable direct agglutinating (IgM) and indirect (IgG) titers. None of 12 selected samples showed monocyte-dependent erythrophagocytosis with or without complement activation. The donor sample causing a hemolytic transfusion reaction and 2 of the 10 patient sera with hemolysin activity showed significant erythrophagocytosis (> 10%) only when complement C3 was activated. The single donor plasma and two sera demonstrating significant erythrophagocytosis had high IgM (≥ 128) and IgG titers (> 1024). The donor plasma anti-A was IgG1, while the patient sera were an IgG3 and an IgG1 plus IgG2.

**Conclusion:**

High anti-A IgM/IgG titers act synergistically to cause significant monocyte erythrophagocytosis by activating complement C3, thus engaging both Fcγ- and CR1-receptors.

## Background

Hemolytic reactions due to minor ABO incompatible transfusions have been documented [1–9] and are most often thought to be associated with high titer anti-A/A,B [6]. Various methods have been used to titer platelet products, and high titer minor ABO incompatible products have been transfused to patients without clinical impact [6, 7].

Antibody-associated extravascular hemolysis is the destruction of red blood cells (RBCs) by resident monocytes/macrophages in spleen and liver [10]. The process is largely governed by IgG and is mainly seen in delayed hemolytic transfusion reactions [11, 12]. Fcγ-receptors present on monocytes/macrophages bind the Fc portion of IgG on sensitized RBCs leading to their phagocytosis [10, 13]. Monocytes/macrophages also possess complement C3b receptors (CR1) on their surface, which can bind and engulf complement coated RBCs. It has been reported that ABO antibody-dependent complement activation alone is insufficient to cause significant erythrophagocytosis [13–15].

A strategy to mitigate extravascular hemolysis based on ABO titer alone may unduly limit the use of platelet products that otherwise pose little risk. In vitro monocyte monolayer assay (MMA) is a traditional, laboratory based testing method that is used to predict the outcome of a transfusion in patients with alloantibodies against RBC antigens [16]. To assess erythrophagocytosis, we used a rapid, automated flow cytometry-based monocyte suspension assay (MSA) in which monocytes and antibody-sensitized fluorescent RBCs are co-incubated in suspension rather than using a glass slide adherent monocyte monolayer. We used anti-A as a model to evaluate IgG and IgM-mediated complement C3b activation and the subsequent influence on monocyte/macrophage erythrophagocytosis. The outcome of this study underscores the need to establish ABO IgM/IgG titers to identify specific risk for hemolytic reactions. Although preliminary in nature, our study provides a framework to free up more units for minor ABO incompatible platelet transfusions. Extravascular hemolysis due to anti-A or anti-B IgG is related to patient factors like ABO zygosity [17], and in the present study, we identified other immune characteristics that contribute to monocyte-mediated erythrophagocytosis. The outcomes of the study have implications on the criteria used to qualify apheresis platelets for minor ABO incompatible transfusions.

## Results

### Anti-A immunoglobulin titers and complement C3b activation

EH-PT had an IgM titer of 256 and an IgG titer of 1024. All 30 donor plasma had IgM titers ≤ 64. Thirteen donor plasma had IgG anti-A titers ≤ 128 while 17 were ≥ 256. Ten out of 30 plasma samples activated complement C3b and were independent of the IgG titer (Table 1) [18]. IgG anti-A titers for the donor plasma causing the minor ABO transfusion reaction and sera H1–H5 are summarized in Table 2.

**Table 1:** IgG/IgM- anti-A titers and complement activation status of plasma samples

No.	Anti-A IgG card	Anti-A IgM card	Anti-C3b/d
*Complement non*-*activating* (*Group 1*)			
Low titer			
1	16	8	0
2	32	16	0
3	128	16	0
4	128	16	0
5	128	8	0
6	128	16	0
7	128	16	0
8	128	8	0
9	128	8	0
High titer			
10	256	32	0
11	256	32	0
12	256	16	0
13	256	16	0
14	256	16	0
15	512	32	0
16	512	32	0
17	512	16	0
18	512	32	0
19	1024	64	0
20	1024	16	0
*Complement activating* (*Group2*)			
Low titer			
21	32	64	1+
22	64	64	2+
23	128	64	1+s
24	128	32	1+
High titer			
25	256	32	1+
26	256	16	1+
27	512	64	1+s
28	512	64	1+s
29	1024	64	1+
30	1024	64	M+

Titer-matched samples were taken from both the groups. Italics rows indicate the samples used in the study (data has published previously in Pandey et al. [18])

**Table 2:** IgG/M anti-A titers, IgG subclass and complement activation status of sera samples

Hemolysin	IgG Anti-A	IgM Anti-A	Anti-A (2-ME treated)	IgG subclass (2-ME treated)	Anti-C3b/d
High titer					
H1	>1024	32	1024	1	W
H2	>1024	32	512	1	W
H3	>1024	128	>1024	1, 2	2+
H4	>1024	256	>1024	3	2+s
H5	>1024	128	>1024	2	1+
Low titer					
H6	32	32	–	–	W
H7	64	8	–	–	M+
H8	128	16	–	–	M+
H9	64	16	–	–	M+
H10	32	32	–	–	0

Hemagglutination grading: 0, no reaction/no agglutination; M+ , RBC agglutinates visible microscopically; W, weak reaction, many RBC tiny agglutinates; 1+ , many small agglutinates; 2+ , many medium sized visible agglutinates, – not done. IgG subclass evaluation was performed only for high titer samples as few of those samples only demonstrated significant (> 5%) erythrophagocytosis

### Erythrophagocytosis

Functional studies were performed with monoclonal BRAD3 anti-D at a concentration of 1:800 and BRAD5 at a concentration of 1:100. BRAD3 sensitization caused significant erythrophagocytosis with all the 3 Rh phenotypes (> 65%) while BRAD5 sensitization caused lower phagocytosis (≤ 6%) (Additional file 1: Figure S1). IVIG inhibited monocyte phagocytosis, with a 50 percent inhibitory concentration (IC50) of 5.3 ± 0.4 µg/mL (Additional file 1: Figure S2). IgG1 (IC50 = 6 µg/mL) and IgG3 (IC50 = 2 µg/mL) inhibited phagocytosis more efficiently than IgG4 (IC50 = 79 µg/mL) and IgG2 (IC50 = 260 µg/mL) (Additional file 1: Figure S3). Effect of diluent on monocyte mediated erythrophagocytosis was measured. Percent erythrophagocytosis of CFDA-SE stained RBCs was observed to remain same (~ 90%) when 1/800 BRAD3 prepared in different diluents 0.2% FBS/PBS, Plasma, and Serum was used for the sensitization of RBCs (Additional file 1: Figure S4).

#### Anti-A-dependent erythrophagocytosis studies

**Fig. 1:**
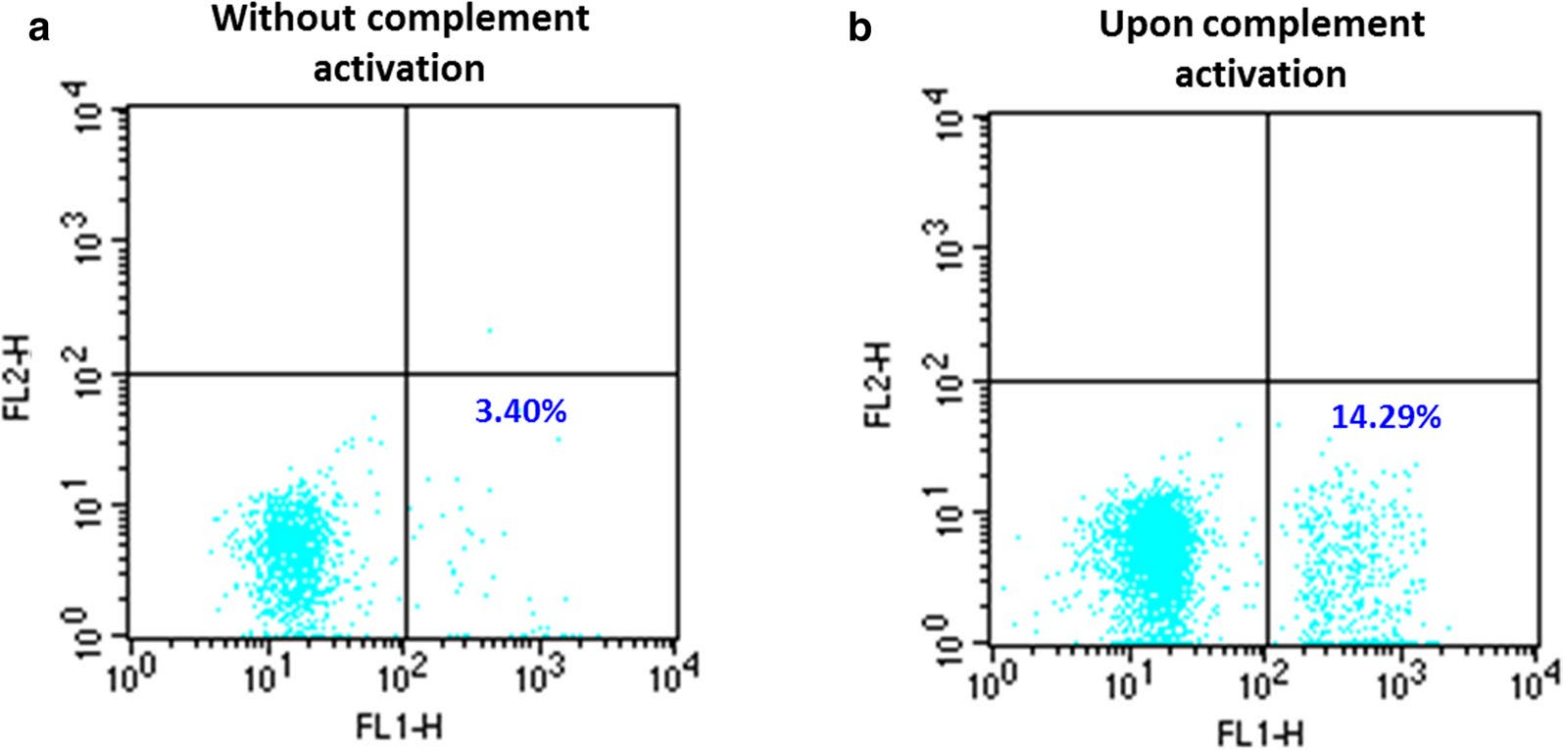
Erythrophagocytosis by plasma from a Group O donor which caused hemolysis in a Group A patient after transfusion of a minor ABO incompatible apheresis platelet unit (EH-PT). **a** No significant phagocytosis was observed when Group A1 + RBCs were coated with the donor plasma without complement activation. **b** A significant increase in phagocytosis was observed upon complement C3b activation

**Fig. 2:**
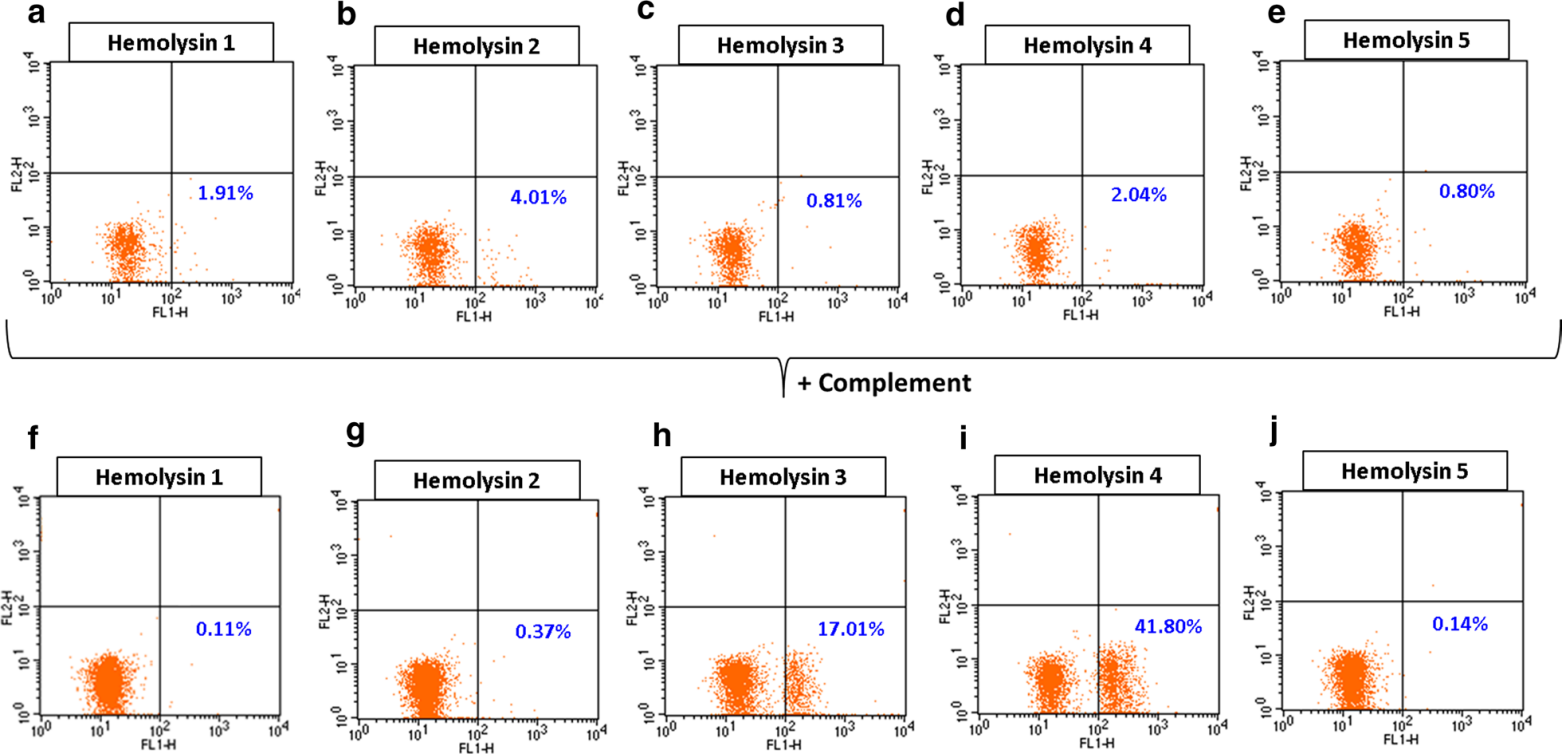
High titer IgG anti-A hemolysins mediated monocyte erythrophagocytosis. **a**–**e** No significant phagocytosis was observed when Group A1 + RBCs were coated with high titer IgG anti-A hemolysin only. **f**, **j** No significant increase in phagocytosis was observed when Group A1 + RBCs were coated with high titer IgG anti-A hemolysin H1 (**f**), H2 (**f**), and H5 (**j**) and fresh Group A serum as a source of complement. A significant increase in phagocytosis was observed upon complement C3b activation for hemolysin H3 (17.01%; **h**) and H4 (41.8%; **i**). Fresh Group A sera was used as a source of complement. RBCs: red blood cells

A selection of 12 donor plasma with low or high anti-A IgG titers had no significant erythrophagocytosis whether complement was activated or not. No significant erythrophagocytosis was observed in EH-PT or any of the patient sera in the absence of complement activation. EH-PT and 2of 10 sera (H3 and H4) showed significant phagocytosis upon complement activation; EH-PT = 14.3% (Fig. 1), H3 = 17.0%, H4 = 41.8% (Fig. 2). Controls consisting of anti-D sensitized RBCs (1:10,000 BRAD3 or 1:800 BRAD5) did not show any significant erythrophagocytosis (≤ 2%) in the absence of complement activation. RBCs coated with complement alone using the anti-A IgM clone demonstrated < 5% phagocytosis. Significant erythrophagocytosis was observed with BRAD3 (30% erythrophagocytosis) and BRAD5 (51% erythrophagocytoisis) when the anti-A IgM clone was added along with complement activation (Table 3).

**Table 3:** Fcγ- and C3b-receptor mediated erythrophagocytosis with MSA controls

Immunoglobulin	Complement source	Phagocytosis (%)
BRAD3 + IgM Anti-A	–	1
BRAD3 + IgM Anti-A	Pooled fresh Group A serum	30
BRAD5 + IgM Anti-A	–	2
BRAD5 + IgM Anti-A	Pooled fresh Group A serum	51
IgM Anti-A	–	1
IgM Anti-A	Pooled fresh Group A serum	3

RBCs coated with complement C3b only showed < 5% phagocytosis. A significant increase in monocyte phagocytosis was observed upon complement C3b activation relative to RBCs sensitized with BRAD3 or BRAD5 only. Dilutions used = 1:10000 BRAD3, 1:800 BRAD5, 1:6000 murine monoclonal anti-A. Group A plasma was used in the place of pooled Group A serum as a negative control

## Discussion

Transfusion of ABO incompatible platelets is an accepted clinical practice. However, IgG and IgM anti-A/B present in the plasma could lead to RBCs destruction [19]. To prevent occurrence of such adverse hemolytic events, mitigation strategies like transfusion of platelets containing low titer antibodies has been adopted [20]. Nonetheless, lack of consensus on a standard method for evaluating anti-A/B titer for incompatible plasma has led to a difficulty in adopting an appropriate cut-off titer. There is little information on the characteristics of ABO antibodies causing hemolysis other than the titer. We studied the plasma from a Group O donor that caused an extravascular transfusion reaction in a Group A recipient (EH-PT), and we correlated the extravascular hemolysis using an in vitro monocyte suspension assay (MSA) that demonstrated both Fcγ and CR1 receptor-dependent erythrophagocytosis [21].

The sensitivity of purified monocytes to phagocytose RBCs in suspension was explored. R2R2 RBCs with the highest copy number of RhD antigen had maximum amount of erythrophagocytosis (Additional file 1: Figure S1). Further, consistent with previous published works on IgG subclass, higher phagocytosis by IgG3 anti-D (clone BRAD3) was observed when compared to IgG1 anti-D (BRAD5) [22]. Also, the pattern of Fcγ receptor blockade by IVIG and IgG subclasses was consistent with the previously published literature [16, 23].

We analyzed a subset of anti-A in plasma from random blood donors as a model of ABO antibody-mediated monocyte erythrophagocytic system and found that no monocyte erythrophagocytosis occurred with high titer IgG anti-A even when complement C3b was activated. We then evaluated selected Group O patient sera with high and low IgG anti-A titers that demonstrated hemolysin activity at low titer, with the possible rationale that immune anti-A would contribute to complement-dependent CR1 erythrophagocytosis. Consistent with the random donor plasma, patient sera demonstrating hemolysin activity with low IgM and low IgG titers did not cause significant erythrophagocytosis whether complement was activated or not (H6–H10, data not shown). Further, no monocyte erythrophagocytosis was observed among the sera with high IgM and IgG titers in the absence of complement activation (Fig. 2a–e). In contrast, significant erythrophagocytosis was observed with high IgM and IgG titers (H3 and H4) when complement was activated (Fig. 2f, j). We were able to mimic the need for complement activation using a murine anti-A IgM clone to activate complement and engage CR1 and a monoclonal IgG3 or IgG1 anti-D to engage Fcγ receptors (Table 3).

Landim et al. [24] saw no correlation between ABO isohemagglutinin titer and hemolysin activity. In addition, they found that using both isohemagglutinin titer and hemolysin activity as exclusion criteria increased the percentage of platelet units which were otherwise found unsuitable for minor ABO incompatible transfusions, and that such an approach lacked clinical support as an implementation strategy. Our in vitro erythrophagocytosis data is in agreement with Landim [24]. High titer IgG and the ability to activate complement do not cause significant erythrophagocytosis, and hemolysin activity is not a predictor of complement-dependent erythrophagocytosis. However, we found that both high titer IgM and IgG along with the ability to activate complement are factors that result in significant erythrophagocytosis. These samples contained subclass IgG1/2 or IgG3 anti-A (Table 2). Both IgG3 and IgG1 have the ability to activate complement and IgG2 can play a significant role in monocyte erythrophagocytosis [25, 26]. Taken together, this preliminary study demonstrates that both high titer complement-activating IgM and IgG anti-A are necessary to engage both Fcγ and CR1 receptors and result in significant erythrophagocytosis.

Since the antigen copy number per RBC is similar between A and B, and anti-A, anti-B, and anti-A,B titers can be elevated, the criteria we defined for significant monocyte erythrophagocytosis should be generally consistent regardless of the ABO antigen. However, our observation with anti-A needs to be confirmed with Group B and A,B. Furthermore, the impact of anti-A1 specific hemolysis was not explored but might be an important factor [27]. The limitations of our study are the relatively low number of samples analyzed and the lack of a method to assess the potential for intravascular hemolysis. Also, we have not evaluated IgG titers ≥ 2048; rare donors with extremely high IgG titers, e.g. > 4000, are likely significant. We did not detect blood donors with both high titer IgM and IgA anti-A in our small survey of 30 plasma. Therefore, we do not know frequency of blood donors with both high-titer complement-activating IgM and high IgG anti-A. However, one donor apheresis platelet unit with a similar profile of high-titer complement-activating IgM and IgG caused clinically significant extravascular hemolysis in a minor ABO incompatible transfusion recipient. In our small survey, 30 donors did not have the anti-A characteristics necessary to induce significant monocyte erythrophagocytosis. A more comprehensive analysis is necessary to determine the frequency of donors with such characteristics and confirm our findings.

## Conclusions

We have identified serologic conditions necessary for significant monocyte erythrocyte phagocytosis that correlated with extravascular hemolysis in a single transfusion recipient. Our analyses suggest minor ABO incompatible platelet transfusions could be limited to those donors with high-titer complement-activating IgM and IgG titers. Among 30 donor plasma tested, 57% had IgG titers > 256 alone. Limiting half of all donors on the basis of an IgG titer cut-off < 250 places unnecessary constraints on inventory management. Furthermore, IgM titers performed at room temperature do not reflect high levels of IgG. A titer strategy that identifies high-titer complement-activating anti-A has the potential to free up more apheresis units for minor ABO incompatible transfusions. A single method alone that identifies either high-titer IgM or IgG ABO antibodies alone appears insufficient to cause significant extravascular erythrophagocytosis. Larger studies correlating antibody titer and monocytes phagocytosis are warranted to confirm these preliminary findings.

## Methods

Plasma containing Group O apheresis platelet product transfused to a Group A patient resulted in an immune transfusion reaction. For the purposes of this study, the effect of anti-A with Group A1 + sensitized RBCs were evaluated from several sources.

### Sample characteristics

The following samples were evaluated in this study: (1) plasma from a single Group O donor that caused extravascular hemolysis in a Group A patient of a minor ABO incompatible apheresis platelet transfusion (EH-PT), (2) random Group O plasma (n = 30) from healthy whole blood donors, and (3) selected Group O sera (n = 10) from outpatients being evaluated for possible ABO incompatible kidney transplantation. The plasma from healthy donors had known IgM/IgG anti-A titers, some with the ability to activate complement as previously published [18]. The serum samples had known IgG anti-A titers and demonstrated anti-A hemolysis at low titer when tested with freshly clotted serum. Samples demonstrating hemolysis were selected since ‘hemolysin activity’ has been associated with ‘immune’ anti-A rather than naturally occurring anti-A [28].

IgM and IgG anti-A titers were evaluated using buffered and anti-IgG gel agglutination cards (Ortho Clinical Diagnostics, Pompano Beach, FL, USA), respectively according to the manufacturer’s instructions. To evaluate the effect of direct agglutination by pentameric IgM, the serum samples with high anti-A titers were treated with 1.1 M 2-mercaptoethanol (2-ME) in a 10:1 ratio at 37 °C for 15 min to assess hemagglutinin titers using buffered and anti-IgG gel agglutination cards. Complement activation of anti-A sera was evaluated at a dilution of 1:100 (vol:vol) according to the previously published protocol [18]. Briefly, a 5% Group A1 + RBC suspension was incubated at 37 °C for 30 min with 200 µL 1:100 serum and 200 µL pooled freshly clotted Group A serum from healthy donors as a source of complement. Sensitized RBCs were washed and checked for complement C3 activation using anti-C3b/d (Gamma-clone, Immucor, Norcross, GA, USA). IgG titers ≥ 256 were deemed ‘high titer’ for the purposes of this publication.

### RBC fluorescent labeling

Group A1 + RBCs were used in the study. Rh-positive (R1r) RBCs were used in initial studies to establish the MSA. A 5% RBC suspension in 0.2% bovine serum albumin/phosphate buffered saline (pH 7.4) was labeled with 6 mM 5(6)-Carboxyfluorescein diacetate N-succinimidyl ester (CFDA-SE; Sigma Aldrich, St. Louis, MO, USA). The labeling reaction was stopped with heat inactivated fetal bovine serum (FBS; Thermo Fisher Scientific, Waltham, MA, USA). The reaction mix was washed and the RBC pellet was resuspended to 5% with 0.2% FBS/PBS (pH 7.4).

### RBC sensitization

Based on initial observations from 30 Group O plasma [18], the samples were divided into 4 groups: (1) low IgG titer, non-complement activating, (2) high IgG titer, non-complement activating, (3) low IgG titer, complement activating, and (4) high IgG titer, complement activating. The patient sera with hemolysin activity were divided into 2 clusters (1) low IgG titer and (2) high IgG titer. Representative donor plasma and patient sera from each cluster (n = 12, highlighted in Table 1) were diluted 1:100 and used to sensitize Group A1 + red cells at 37 °C for 30 min with and without fresh Group A serum. All sensitized RBCs were washed and checked for IgG sensitization and complement C3 activation using anti-IgG and anti-C3b/d (Gamma-clone, Immucor, Norcross, GA, USA), respectively. An aliquot of sensitized, washed RBCs resuspended at 1% in monocyte media (CO_2_ independent media containing 10% FBS and 1% l-Glutamine; Thermo Fisher Scientific, Waltham, MA, USA) was used to evaluate monocyte erythrophagocytosis.

To monitor the performance of MSA, control experiments were performed as described (see results in supplemental files). *Controls:* Mock controls consisting of IgG and C3b sensitized RBCs were prepared using a 1:10,000 dilution of anti-D IgG3 (clone BRAD3; American Research Products Inc., Waltham, MA, USA) or a 1:800 dilution of anti-D IgG1 (clone BRAD5; American Research Products Inc.) with or without a 1:6000 dilution of murine monoclonal IgM anti-A plus fresh Group A serum as a source of complement C3. Controls were prepared also without fresh Group A serum. When tested alone, BRAD3, BRAD5, plus the murine IgM anti-A clone did not demonstrate monocyte erythrophagocytosis; a fresh source of pooled A serum was needed to activate complement and cause significant erythrophagocytosis. The effect of the diluent used in the MSA was evaluated using BRAD3 diluted in human AB serum and plasma. The results were compared to the MSA for RBCs suspended in 0.2% BFBS/PBS.

### Erythrophagocytosis: monocyte suspension assay (MSA)

**Fig. 3:**
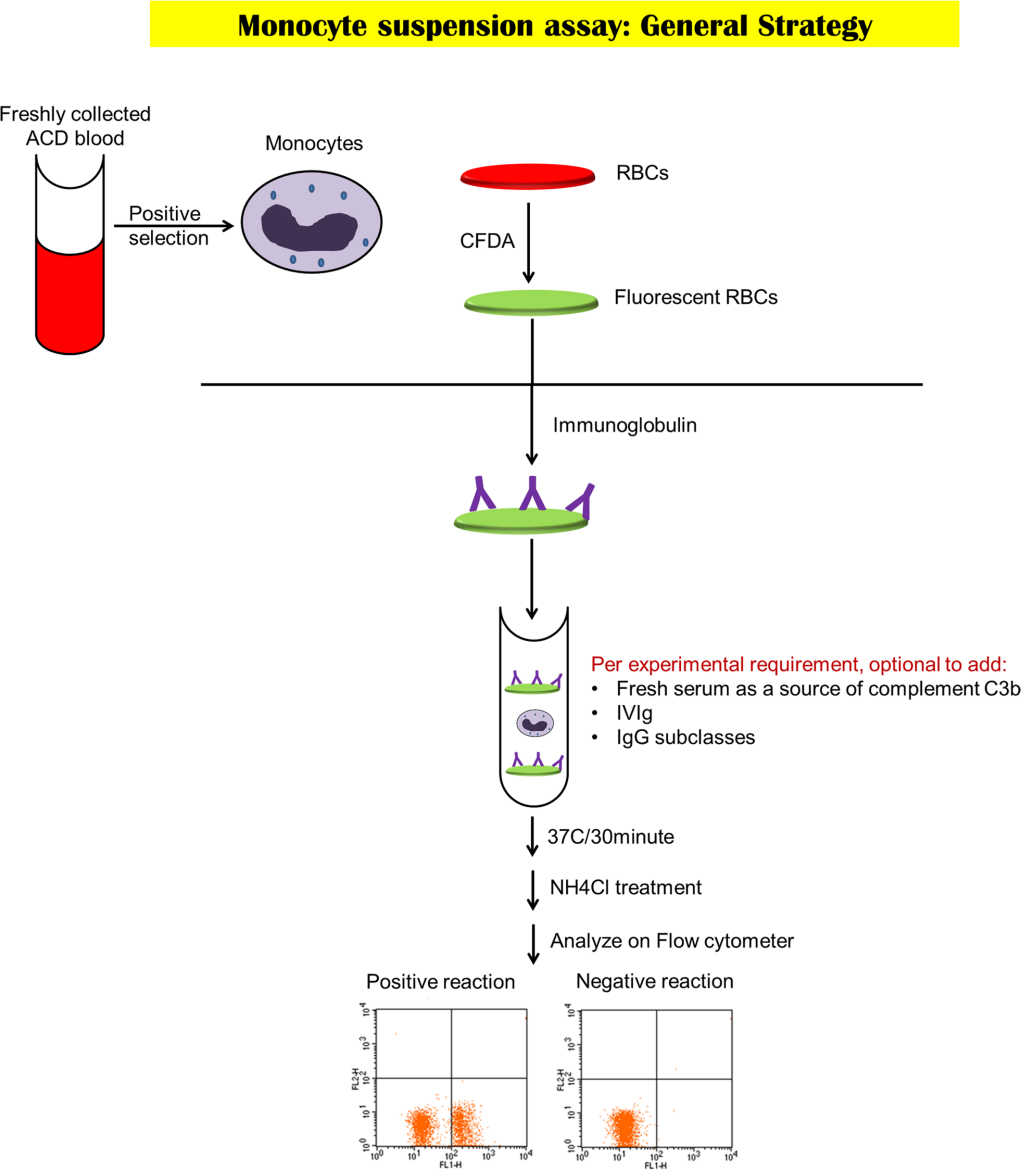
Study design. The general strategy of the monocyte suspension assay (MSA)

Peripheral blood mononuclear cells (PBMCs) from a healthy donor were isolated from freshly collected ACD whole blood using density gradient medium (Stemcell Technologies, Vancouver, BC, Canada). Monocytes were purified from PBMCs by immunomagnetic CD14-positive selection (Stemcell Technologies, Vancouver, BC, Canada), frozen in 10% dimethyl sulfoxide/FBS (Thermo Fisher Scientific, Waltham, MA, USA) at − 80 °C and then thawed on the day of use. Before performing MSA, monocytes were thawed rapidly at 37 °C, washed once using saline, and resuspended in monocyte media. The cells were counted and assessed for viability using counting beads (Thermo Fisher Scientific, Waltham, MA, USA) and propidium iodide (Stemcell Technologies, Vancouver, BC, Canada). To measure phagocytosis, 50 µL monocytes (0.5–1 × 10^5^) were incubated with 60 µL 1% sensitized RBCs in 190 µL monocyte media (total reaction volume = 300 µL) in the dark at 37 °C for 30 min. Fluorescent labeled unsensitized RBCs served to evaluate as a negative control for erythrophagocytosis. Phagocytosis was stopped by one wash with saline and non-phagocytosed RBCs were lysed with 4 °C ammonium chloride for 5 min followed by one wash in saline. Flow cytometry was performed on FACSCalibur (BD Biosciences, CA). Data was analyzed using CellQuest Pro software (BD Biosciences, Franklin Lakes, NJ, USA). Increase in fluorescence in the FL1 channel (517 nm) was recorded as a positive signal for phagocytosis (Fig. 3). The phagocytic function of monocytes in suspension was evaluated by showing the effect of (1) Rh D antigen dosage on erythrophagocytosis using BRAD3 and BRAD5, (2) the dose–response inhibition by IVIG, and (3) the variable inhibitory effect of IgG subclasses (Additional file 1). Erythrophagocytosis ≥ 5% was deemed a significant observation based on reproducibility studies and the values obtained for the negative controls.

### IgG subclass

EH-PT and sera with high IgG titers (N = 5) were evaluated for IgG subclass after 2-ME treatment. 2-ME treatment was needed as high IgM in the samples was causing direct agglutination thereby obscuring the evaluation of IgG subclass at 1:50 dilution. A 1:50 diluted 2-ME treated sera were used to sensitize A1 + RBCs at 37 °C for 30 min. The reactions were washed and evaluated for the subclass using 1:50 diluted Anti-IgG1, -IgG2, -IgG3, and -IgG4 (Sigma Aldrich, St. Louis, MO, USA).

## Supplementary information

**Additional file 1.** Monocyte suspension assay supplemental Figures S1–S4.

## Data Availability

All data generated and analyzed during this study are included in this published article and its additional files.

## Abbreviations

2-ME: 2-Mercaptoethanol
CFDA-SE: Carboxyfluorescein diacetate N-succinimidyl ester
EH-PT: Extravascular hemolysis-platelet transfusion
FBS/PBS: Fetal bovine serum/phosphate buffered saline
Fc: Fragment crystallizable
IgM/G: Immunoglobulin G/M
MMA: Monocyte monolayer assay
MSA: Monocyte suspension assay
RBC: Red blood cell
