# Supplementary material for: Complement activating ABO anti-A IgM/IgG act synergistically to cause erythrophagocytosis: implications among minor ABO incompatible transfusions
Source: J Transl Med. 2020 May 28;18:216. doi: 10.1186/s12967-020-02378-w (PMC7257204; doi:10.1186/s12967-020-02378-w)
Supplement: Supplementary file 1 — Additional file 1. Monocyte suspension assay supplemental Figures S1–S4. [file 12967_2020_2378_MOESM1_ESM.docx]

**Additional Figure S1**


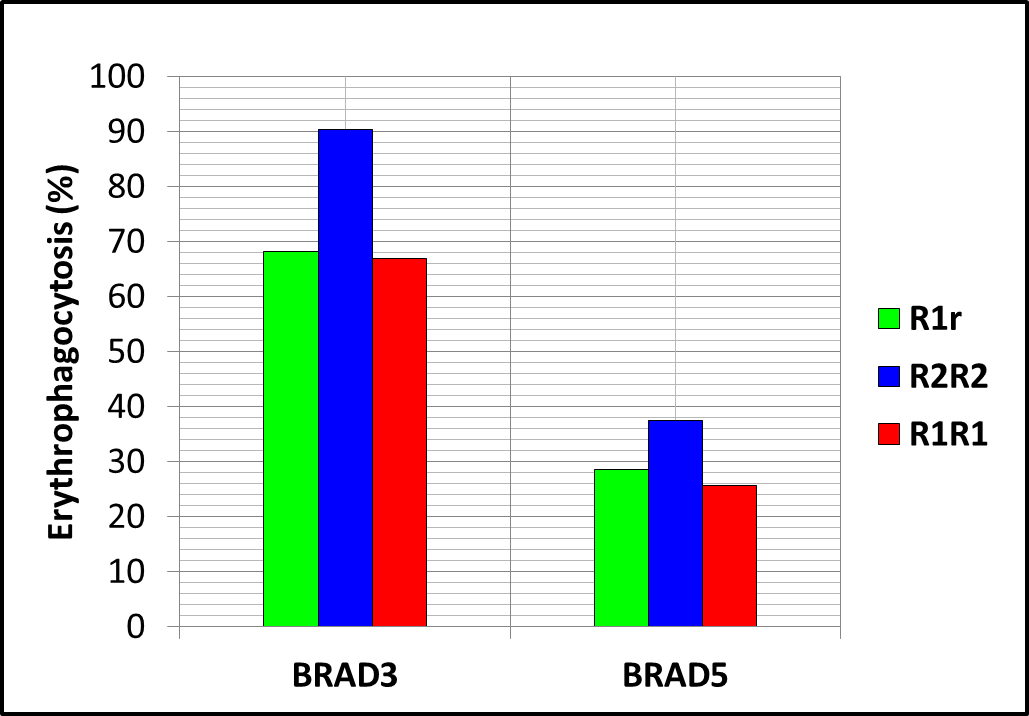


**Variation in phagocytosis per Rh D density.** CFDA-SE labeled Group O+ RBCs (R2R2, R1R1, and R1r) were sensitized with saturating dilutions of RhD IgG3 (BRAD3, 1:800) and IgG1 (BRAD5, 1:100) at 37C for 30 minutes and erythrophagocytosis was performed as detailed in Material and Methods section of the manuscript. IAT anti-IgG for 1:800 BRAD3 or 1:100 BRAD5 sensitized RBCs ≥2+. Monocyte phagocytosis was observed to be proportional to the density of Rh D antigen on the surface of RBCs. **A)** BRAD3 sensitization: Monocytes phagocytosed BRAD3 sensitized R2R2 RBCs (90%) more than R1R1 (67%) and R1r (68%). **B)** BRAD5 sensitization: Monocytes phagocytosed BRAD5 sensitized R2R2 RBCs (38%) more than R1R1 (26%) and R1r (29%). IAT: indirect antiglobulin test, RBCs: red blood cells. A 1:800 BRAD3 and 1:100 BRAD5 dilutions of anti D IgGs was chosen as it gave a hemagglutination score of ≥2+s, needed for a phagocytic index ≥80.^18^

**Additional Figure S2**


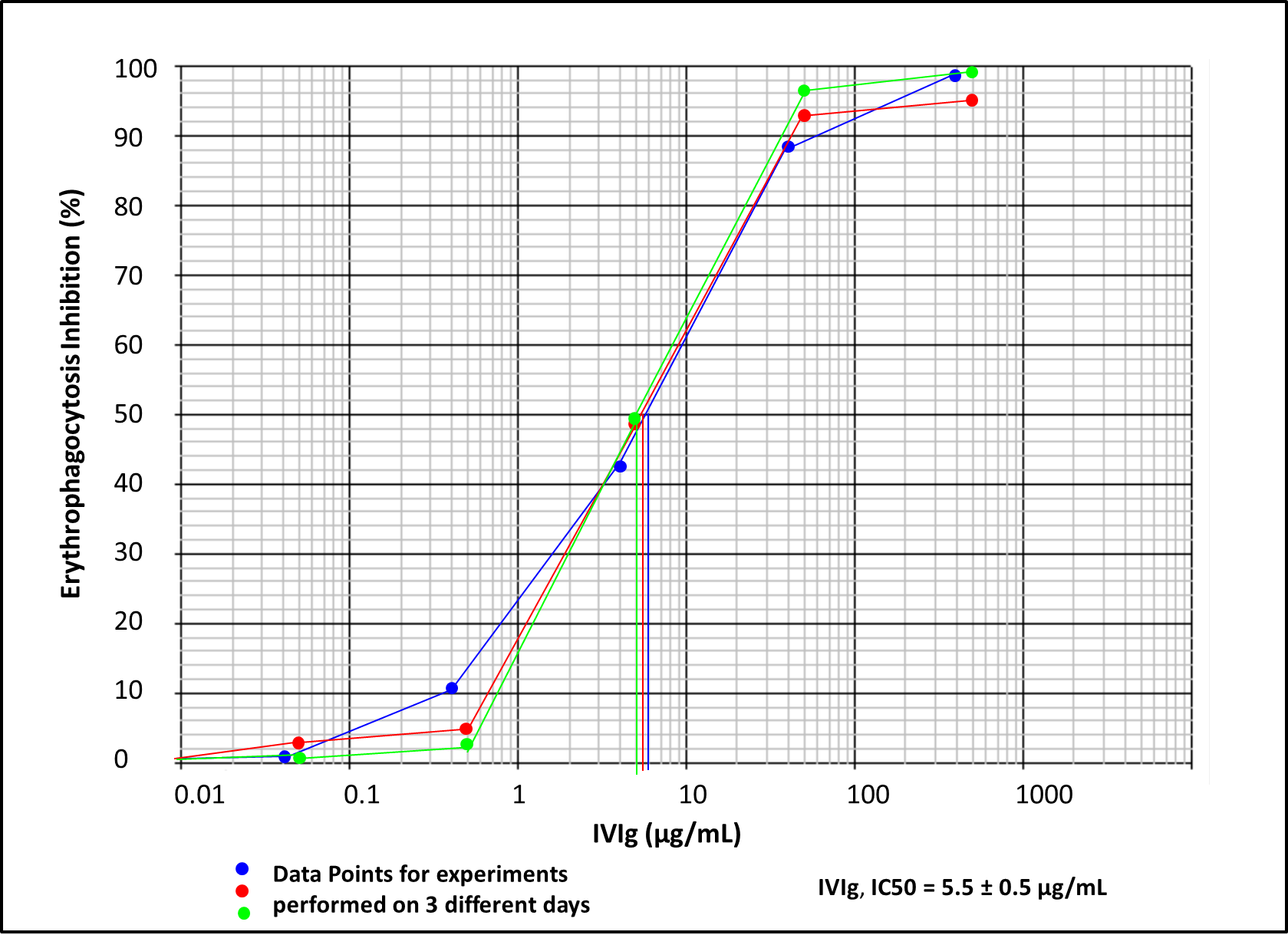


**Inhibition of FcγR mediated phagocytosis by IVIG.** IVIG inhibition assay was performed 3 times using the same source of monocytes. CFDA-SE labeled, BRAD3 (1:800) sensitized Group O R2R2 RBCs were incubated with monocytes and IVIG at the final concentrations indicated in the graph. IVIG inhibited FcγR mediated phagocytosis in a dose dependent fashion with an IC50 = 5.5 ± 0.5 µg/mL (n = 3). IC50: IVIG concentration at which erythrophagocytosis was reduced to 50%, IVIG: intravenous immunoglobulin, RBCs: red blood cells.

**Additional Figure S3**


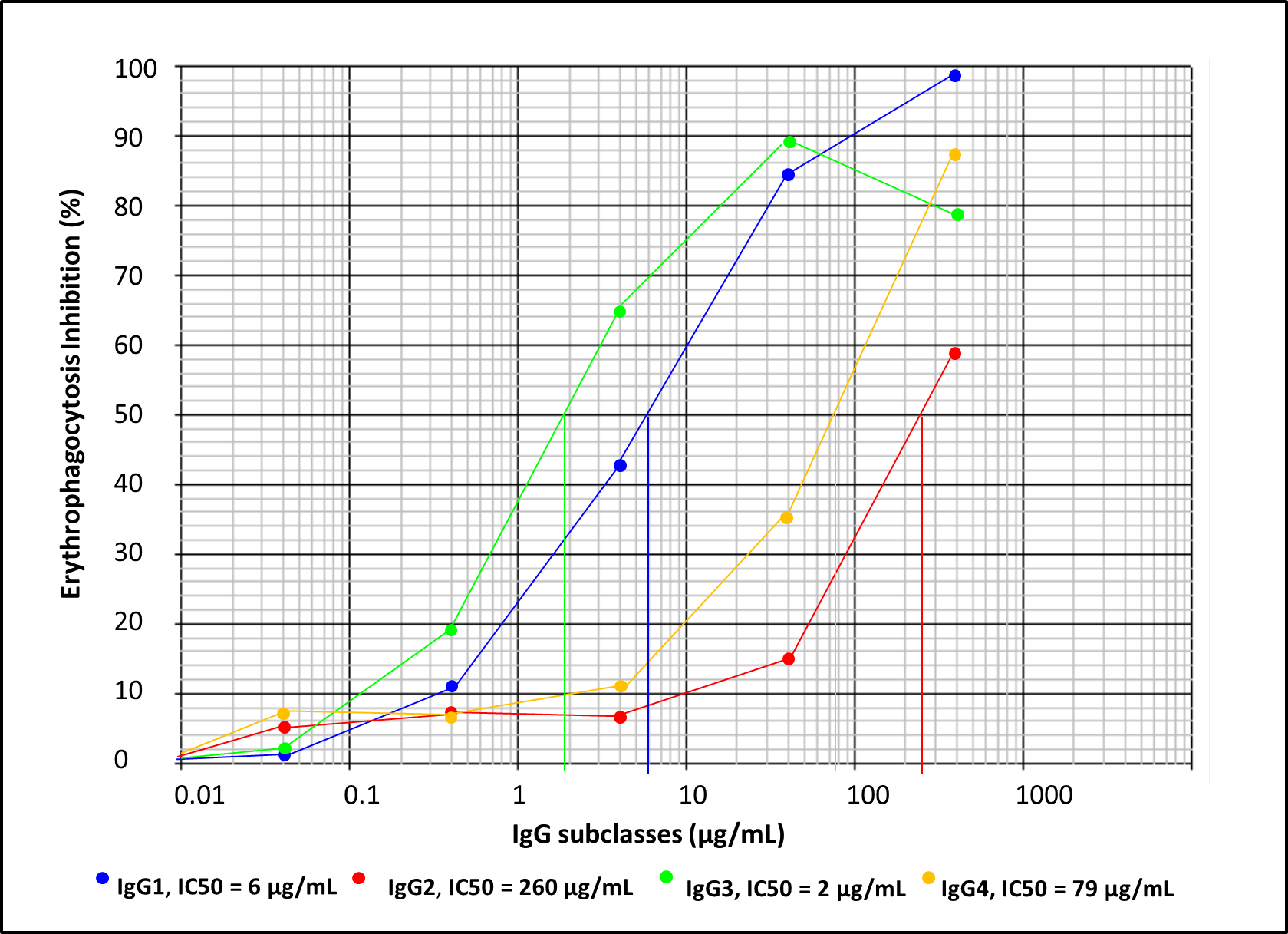


**Inhibition of FcγR mediated phagocytosis by IgG subclasses.** IgG subclass inhibition assay was performed, in which CFDA-SE labeled, BRAD3 (1:800) sensitized Group O R2R2 RBCs were incubated with monocytes and IgG subclasses at the final concentrations indicated in the graph. IgG subclasses inhibited FcγR mediated phagocytosis in a dose dependent fashion. IgG1 and IgG3 inhibited 50% erythrophagocytosis (IC50) at lower concentrations than IgG2 and IgG4. IC: inhibitory concentration, RBCs: red blood cells.

**Additional Figure S4**


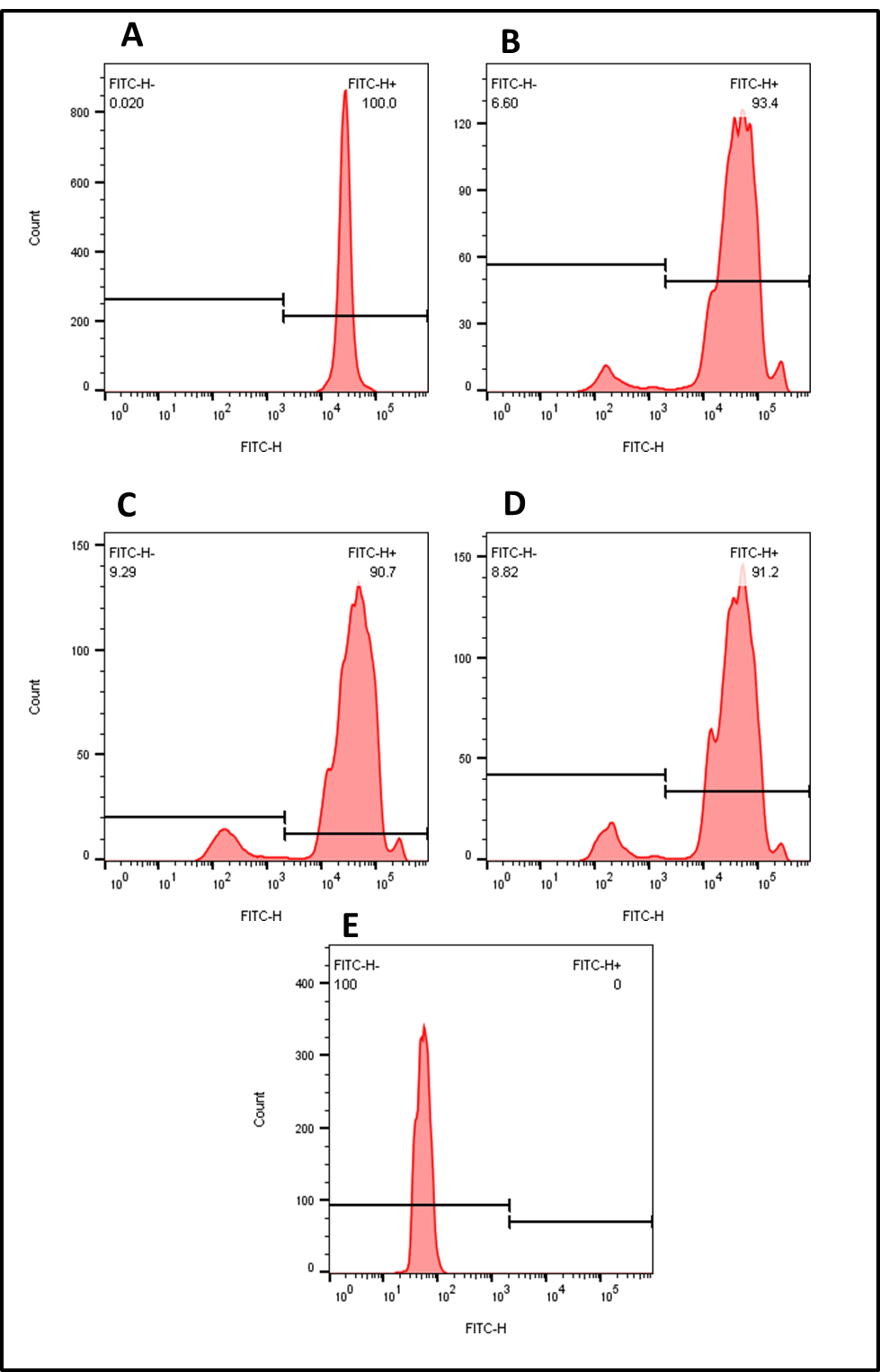


**Effect of diluent on monocyte mediated erythrophagocytosis.** Percent erythrophagocytosis of CFDA-SE stained RBCs (**A**) was observed to remain similar when 1/800 BRAD3 prepared in different diluents 0.2%BSA/PBS (**B**), Plasma (**C**), and Serum (**D**) was used for the sensitization of RBCs. Control (**E**): Un-opsonized RBCs incubated with monocytes for the erythrophagocytosis. RBCs: red blood cells.
